# Driveline Features as Risk Factor for Infection in Left Ventricular Assist Devices: Meta-Analysis and Experimental Tests

**DOI:** 10.3389/fcvm.2021.784208

**Published:** 2021-12-16

**Authors:** Melanie Kranzl, Martin Stoiber, Anne-Kristin Schaefer, Julia Riebandt, Dominik Wiedemann, Christiane Marko, Günther Laufer, Daniel Zimpfer, Heinrich Schima, Thomas Schlöglhofer

**Affiliations:** ^1^Department of Cardiac Surgery, Medical University of Vienna, Vienna, Austria; ^2^Center for Medical Physics and Biomedical Engineering, Medical University of Vienna, Vienna, Austria; ^3^Ludwig-Boltzmann-Institute for Cardiovascular Research, Vienna, Austria

**Keywords:** left ventricular assist device (LVAD), mechanical features, risk factors, driveline infection, mechanical circulatory support (MCS)

## Abstract

**Background:** Risk factors for driveline infection (DLI) in patients with left ventricular assist devices are multifactorial. The aim of this study was to analyze the correlation between mechanical driveline features and DLI occurrence.

**Methods:** A meta-analysis was conducted that included studies reporting DLI rates at 6 months after implantation of any of three contemporary devices (HVAD with Pellethane or Carbothane driveline, HeartMate II, and HeartMate 3). Further, outer driveline diameter measurements and *ex-vivo* experimental three-point bending and torsion tests were performed to compare the stiffness of the four different driveline types.

**Results:** 21 studies with 5,393 patients were included in the meta-analysis. The mean weighted DLI rates ranged from 7.2% (HeartMate II) to 11.9% (HeartMate 3). The HeartMate II driveline had a significantly lower maximal bending force (Load_max_) (4.52 ± 0.19 N) compared to the Carbothane HVAD (8.50 ± 0.08 N), the HeartMate 3 (11.08 ± 0.3 N), and the Pellethane HVAD driveline (15.55 ± 0.14 N) (*p* < 0.001). The maximal torque (Torque_max_) of the HeartMate II [41.44 (12.61) mNm] and the Carbothane HVAD driveline [46.06 (3.78) mNm] were significantly lower than Torque_max_ of the Pellethane HVAD [46.06 (3.78) mNm] and the HeartMate 3 [95.63 (26.60) mNm] driveline (*p* < 0.001). The driveline of the HeartMate 3 had the largest outer diameter [6.60 (0.58) mm]. A relationship between the mean weighted DLI rate and mechanical driveline features (Torque_max_) was found, as the the HeartMate II driveline had the lowest Torque_max_ and lowest DLI rate, whereas the HeartMate 3 driveline had the highest Torque_max_ and highest DLI rate.

**Conclusions:** Device-specific mechanical driveline features are an additional modifiable risk factor for DLI and may influence clinical outcomes of LVAD patients.

## Introduction

Heart failure remains among the main causes of morbidity and mortality worldwide with an increasing prevalence (1, 2). In recent years, left ventricular assist devices (LVADs) have become an established therapeutic option for end-stage heart failure (3) to support the circulation until myocardial recovery, as bridge to transplant, or as long-term destination therapy (DT) (4). Although LVAD recipients have excellent survival rates, postoperative adverse events can lead to impaired quality of life. The most common adverse events in the early and late periods after continuous flow LVAD implant are major infections (5). An infection rate of 9.1% in the first 3 months after LVAD implantation has been previously reported for pump-related percutaneous driveline infection (DLI) (6), that can lead to pain at the driveline exit site (DLES), an increase of medical costs, and even to stroke (7–9). Consequently, DLI is further the primary cause of readmission in LVAD patients (4). The development of DLI is multifactorial, with several reported risk factors such as increased body mass index (BMI) (10–13), history of diabetes mellitus (DM) (10), and an exposed velour (10, 14–16). The probability of developing a DLI seems to rise with the duration of LVAD support (17–19) and reaches a peak 6 months after implantation. This could be related to the increased activity of patients after hospital discharge (20) and the associated increase of trauma at the DLES, which was previously reported as one of the major initiators for DLI (21). Bending or torsion of the driveline is common during daily activity, e.g., changing of clothes, light exercises, or turning around while sleeping, which could lead to trauma at the DLES (4, 21) and rigid materials and large diameters of the driveline could exacerbate this problem. However, there is only limited knowledge about how driveline features such as diameter and stiffness of contemporary devices affect DLI occurrence. Therefore, this study aims to quantify and compare device-specific mechanical driveline properties of three LVADs with four different drivelines and to correlate them with DLI occurrence.

## Materials and Methods

### Meta-Analysis

This meta-analysis is in accordance with the Preferred Reporting Items for Systematic Reviews and Meta-Analyses (PRISMA) statement. (22).

#### Data Source and Search Strategy

**Figure 1:**
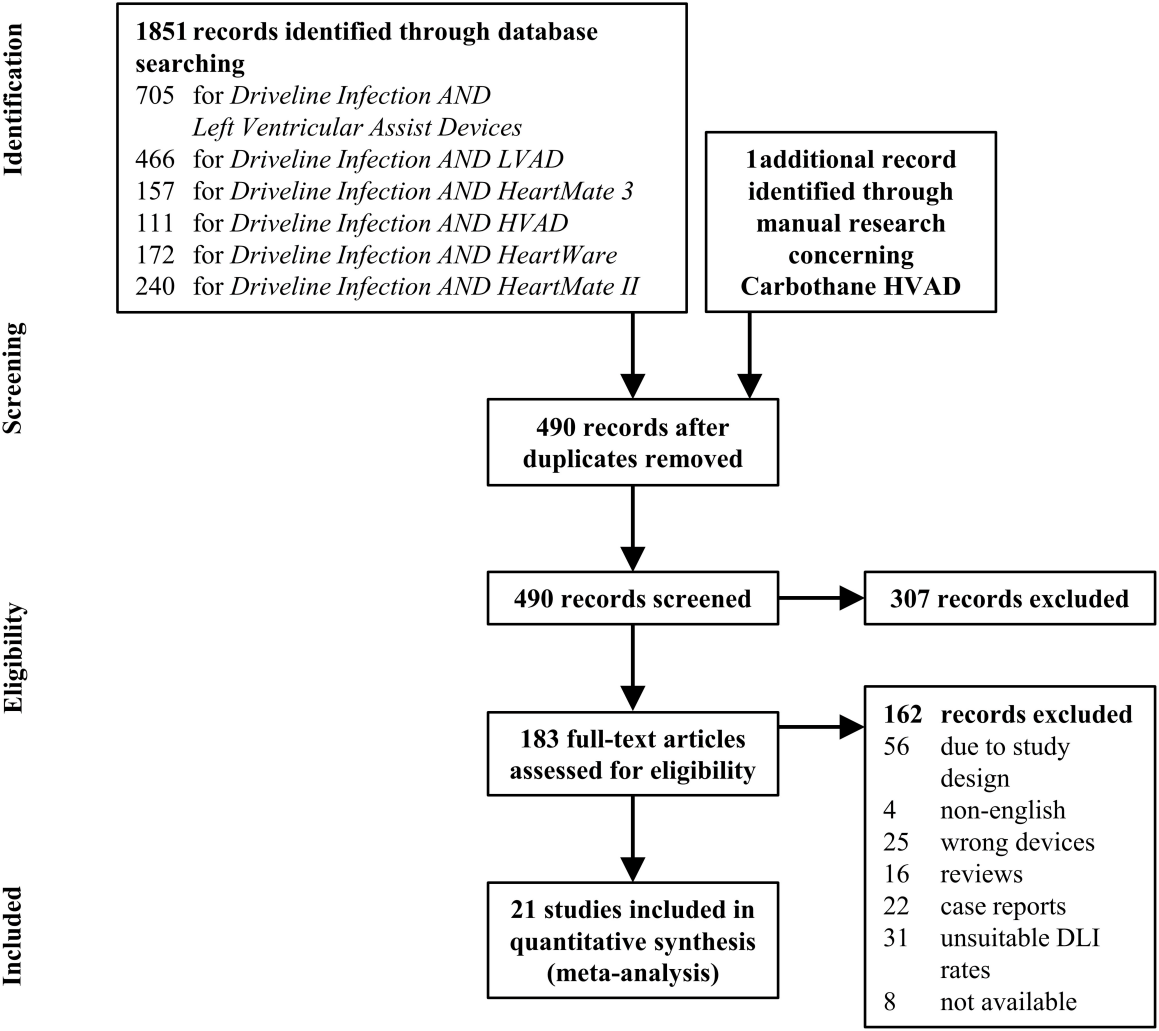
Flow diagram summarizing the systematic research process. DLI, driveline infection.

Two independent reviewers used the databases PubMed and SCOPUS in October 2021 with the search terms “Driveline Infection AND Left Ventricular Assist Devices,” “Driveline Infection AND LVAD,” “Driveline infection AND HeartMate 3,” “Driveline infection AND HVAD,” “Driveline infection AND HeartWare,” and “Driveline Infection AND HeartMate II” to identify studies assessing DLI data of LVAD patients (Figure 1). Since the HVAD Carbothane driveline did not receive FDA approval until 2019 (23), and no studies were found in the database, an additional manual research was performed. The literature search was not limited to the strict PICO format, as this would likely have excluded relevant articles, particularly retrospective cohort studies without a control group.

#### Study Selection and Data Extraction

The outcomes of interest were either a numeric DLI rate at 6 months or a freedom from DLI Kaplan-Meier curve of at least one of the three devices and the sample size. Exclusion criteria included case reports, review articles, non-English articles, records with wrong devices and records with unsuitable DLI rates (e.g., DLI rates stated as events per patient years). Extracted data included the study period, study design, the device, the DLI rate, the cohort's Interagency Registry for Mechanically Assisted Circulatory Support (INTERMACS) classification, DM, age, BMI, DT indication, gender, and implant technique characteristics. The WebPlotDigitizer (Version 4.4, Ankit Rohatgi, 2020) was used to extract the 6 months DLI rate from the Kaplan-Meier curve. A random-effects model was used and for each device type, the extracted DLI rates were weighted with the Schmidt-Hunter method depending on their sample size and used to calculate a mean weighted DLI rate. The evaluation, organization, and analysis of suitable literature sources were done using the software Review Manager (RevMan) (Version 5.4.1, The Cochrane Collaboration, 2020).

#### Study Quality Assessment

Studies were assessed for methodologic quality using the risk of bias tool described in the Cochrane Handbook for Systematic Reviews (24). This tool enables subjective assessment of bias across six domains, including selection, performance, attrition, detection, and reporting.

### Experimental Driveline Analysis

#### Sample Selection

An assortment of new and clinically used driveline samples without velour cover were analyzed. Eleven Pellethane HVAD (Medtronic Inc, Minneapolis, MN, USA), eleven Carbothane HVAD (Medtronic Inc), two HeartMate II (Abbott Inc, Chicago, IL, USA), and six HeartMate 3 (Abbott Inc) were used, and all measurements were repeated five times for each driveline specimen.

#### Three-Point Bending Test

**Figure 2:**
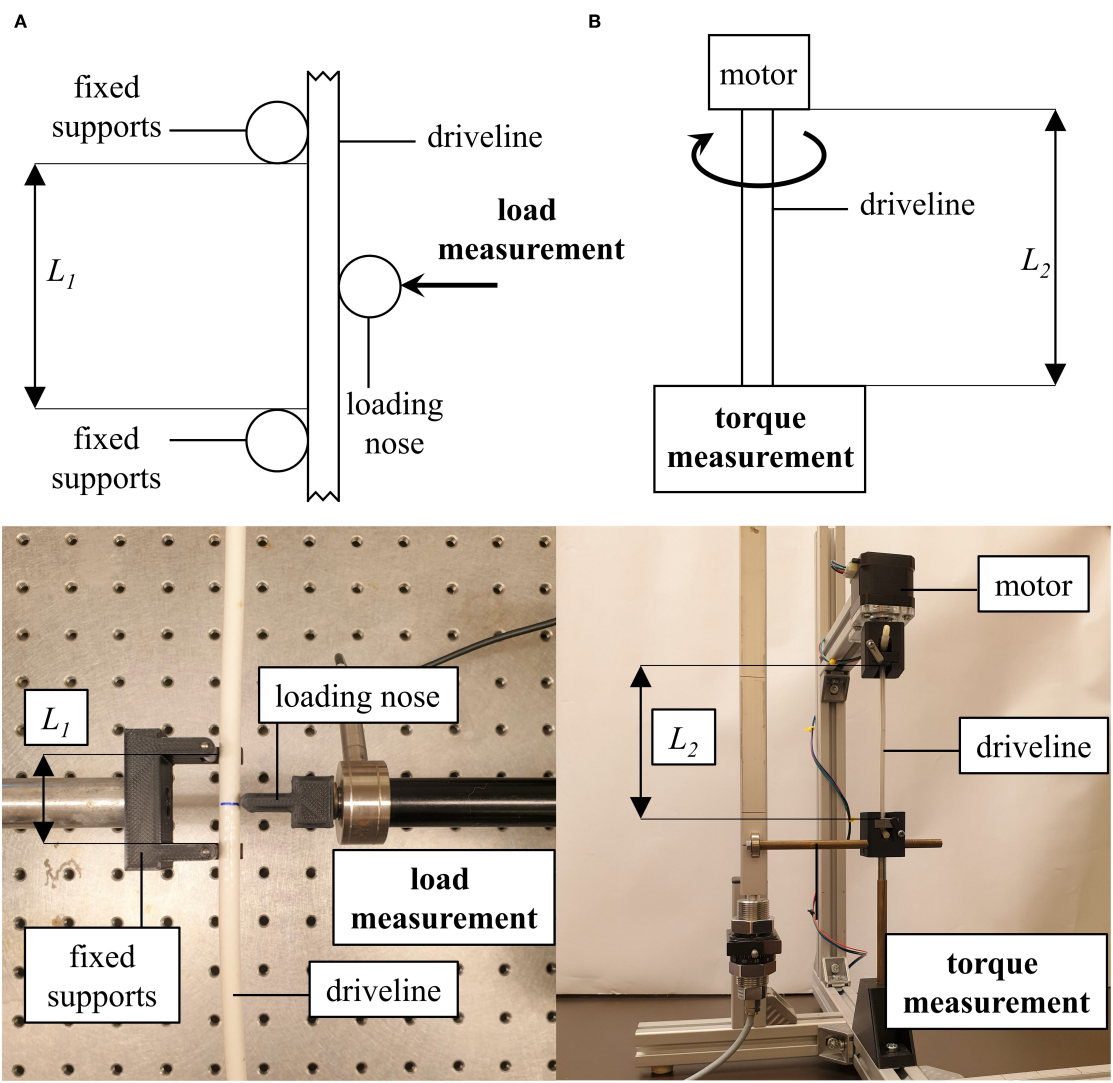
Measurement setup of the three-point bending **(A)** and torsion test **(B)**. (L_1_) support span, (L_2_) free length.

An experimental three-point bending test (Figure 2A) was conducted, based on the standard EN ISO 178:2019 08 01 (25) using a BOSE® LM1 ElectroForce test bench system (Bose Corp. MN, USA) with an integrated displacement transducer. A 3D-printed design with a support span of 30 mm was used, with the radii of the supports and the loading nose being 2.5 mm. A 225 *n* load cell Type WMC-50-543 (Bose Corp. MN, USA) was mounted in line with the motor shaft. The measurement process was performed with the software WinTest® (Version: 7.1.2014- 04.04, Bose Corp. MN, USA) allowing movements of the linear motor and simultaneous recording of time, load, and displacement. The drivelines were bent to a total displacement of 12 mm with a bending velocity of 1.5 mm/s to measure the maximal bending force Load_max_.

#### Torsion Test

The torsion test (Figure 2B) was modified from the standards EN 50289-3-10:2005 11 01 (26) and EN ISO 25539- 2:2019 06 01 (27). The drivelines were clamped vertically into a custom-made torsion testing apparatus with a free length of 12 cm. An Arduino Uno R3 (Adafruit Industries, New York, USA) was used to operate a 42SHDC3025-24B stepper motor (Anet Technology Co., Ltd., Shenzhen, CHN) to twist the driveline (720°) with an angular velocity of 100°/s. An iron bar was attached at the lower end of the driveline which was mounted in a tube as a duct and a lever arm with 10 cm was attached. When the stepper motor twisted the driveline, the lever arm pressed against a bar mounted on a RFS® 150 XY sensor (Honigmann Industrielle Elektronik GmbH, Gevelsberg, DEU) to measure the maximal torque (Torque_max_). The DS1103 PPC Controller Board and the software ControlDesk (Version: 5.0, 2013, dSPACE GmbH, Paderborn, DEU) were used for the simultaneous recording of time and torque.

### Statistical Analysis

Descriptive statistics are reported as mean ± standard deviation for normally distributed continuous variables and as median and interquartile range (IQR) for non-normally distributed values. Normal distribution was assessed by the Shapiro-Wilk test. One-way analysis of variance (ANOVA) or Kruskal-Wallis tests were used to test continuous variables (Load_max_ and Torque_max_) between the four driveline groups. When statistical significance was found (*p* < 0.05), *post-hoc* analyses were performed. Therefore, a Levene's test was used to check the homogeneity of variance with a significance level of *p* < 0.05. If homogeneity of variance was present, a Bonferroni-test was performed, otherwise a Games Howell test for normally distributed groups was used. In both cases, the significance level was set to *p* < 0.05. For non-normally distributed values, a pairwise comparison was performed with a Bonferroni correction, and the significance level was set at *p* = 0.0125. Statistical analysis was performed by SPSS for Windows Release 26.0.0 (SPSS Inc, Chicago, IL, USA) and MATLAB R2020a (The MathWorks Inc, Natick, MA, USA).

## Results

### Meta-Analysis

**Figure 3:**
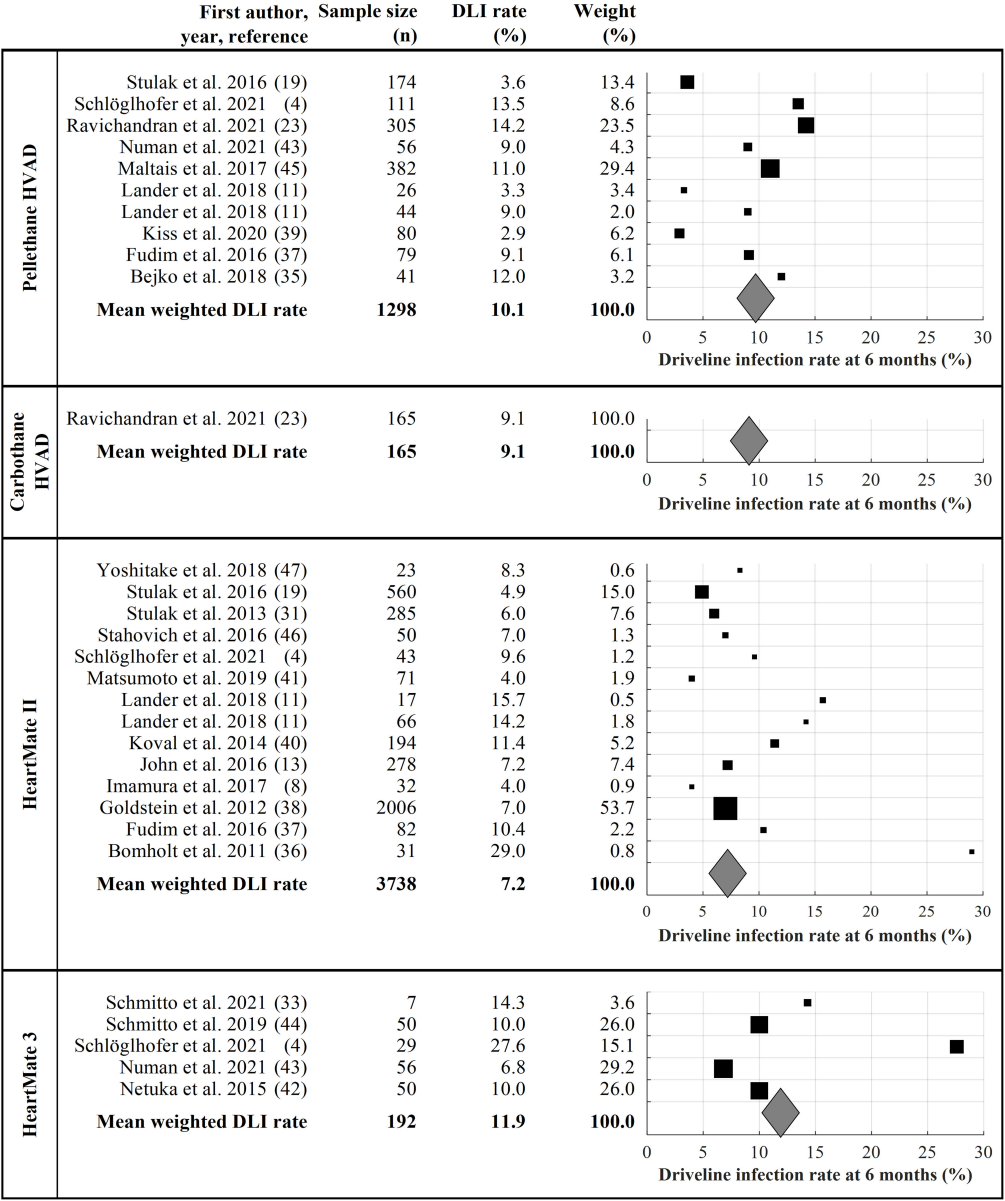
Summary of all included and analyzed studies. The black marks represent the DLI of the study, the size of the black marks refers to the number of included patients. The mean weighted driveline infection rate for each device is represented by gray diamonds. DLI, driveline infection rate.

Of the 490 full-text articles screened, *n* = 20 articles fulfilled the inclusion criteria and reported DLI rates at 6 months following LVAD implantation in one or more of the included device types (Figure 1). Manual search on DLI rates of patients supported with the Carbothane HVAD driveline revealed *n* = 1 abstract. In total, 5,393 patients were included in the final meta-analysis. The most studies (*n* = 14) and included patients (*n* = 3738) were identified for the HeartMate II. The mean weighted DLI rates ranged from 7.2% (HeartMate II) to 11.9% (HeartMate 3). The final 21 articles, including the DLI rate after 6 months for each study and the mean weighted DLI rate for each driveline type are summarized in Figure 3. The overall mean weighted DLI rate including all studies was 8.1%. Of the included studies with reported patient characteristics, INTERMACS Class 1 ranged from 0 to 41.0%, age was between 38 ± 13 and 62.4 ± 8.3, BMI ranged from 20.4 ± 3.5 and 29.7 ± 6.23, 13.0% to 43.8% suffered from DM, 11% to 100% received their LVAD as DT, and 65.4% to 93% were male (see Supplementary Table 1).

Assessments of study quality and risk of bias are summarized in Supplementary Table 2. In the fast majority of studies, a low risk for performance (85.7%), detection (100%) and reporting (90.5%) bias was found. Moderate selection bias was more common (23.8%), whereas the attrition bias was rated as low (66.6%) or unclear (23.8%) in most studies.

### Experimental Driveline Analysis

**Figure 4:**
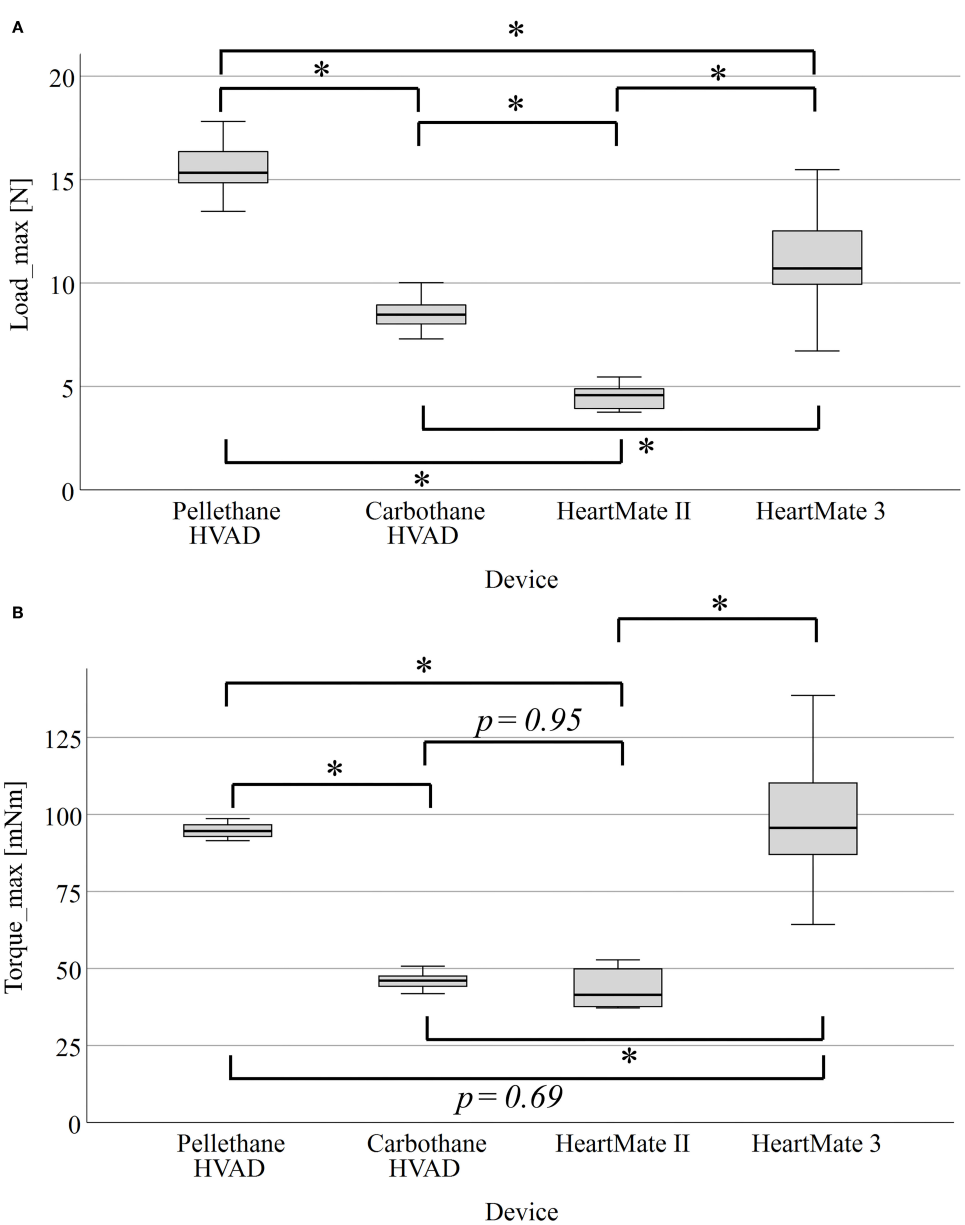
Boxplots of Load_max_ of the three-point bending test of four different driveline types **(A)** and Torque_max_ of the torsion tests **(B)**. *p < 0.001. Load_max_, maximal bending force; Torque_max_, maximal torque.

In total, 30 driveline samples were analyzed and Table 1 summarizes their mechanical features. Among the four observed driveline types, Carbothane HVAD and Pellethane HVAD had the smallest diameter, with 4.8 (0.0) mm. The least rigid driveline in the three-point bending test was the HeartMate II (Load_max_ = 4.27 ± 0.07 N), whereas the Pellethane HVAD driveline had a significantly higher Load_max_ = 13.56 ± 0.08 *n* (*p* < 0.001). The stiffness of each driveline type is shown in Figure 4A. Significant differences (*p* < 0.001) were found between all groups. In the torsion tests (Figure 4B), the HeartMate II driveline had the lowest Torque_max_ [41.44 (12.61) mNm] and the HeartMate 3 driveline had the highest Torque_max_ [95.63 (26.60) mNm]. Further, the HeartMate 3 driveline Torque_max_ was significantly higher (*p* < 0.0125) compared to the Carbothane HVAD and the HeartMate II drivelines. Comparable results were found between the Carbothane HVAD and the HeartMate II (*p* = 0.95) as well as between the Pellethane HVAD and the HeartMate 3 drivelines (*p* = 0.69).

**Table 1:** Summary of the mechanical features of the analyzed drivelines.

	**Load_**max**_ [N]**	**Torque_**max**_ [mNm]**	**Diameter [mm]**
Pellethane HVAD	15.55 ± 0.14	94.62 (3.89)	4.8 (0.0)
Carbothane HVAD	8.50 ± 0.08	46.06 (3.78)	4.8 (0.0)
HeartMate II	4.52 ± 0.19	41.44 (12.61)	6.0 (0.0)
HeartMate 3	11.08 ± 0.30	95.63 (26.6)	6.6 (0.58)

*Values are either presented as mean ± standard deviation for normally distributed groups or as median (interquartile range) for non-normally distributed groups. Load_max_, maximal bending force; Torque_max_, maximal torque*.

### Relationship of DLI Rates and Driveline Features

**Figure 5:**
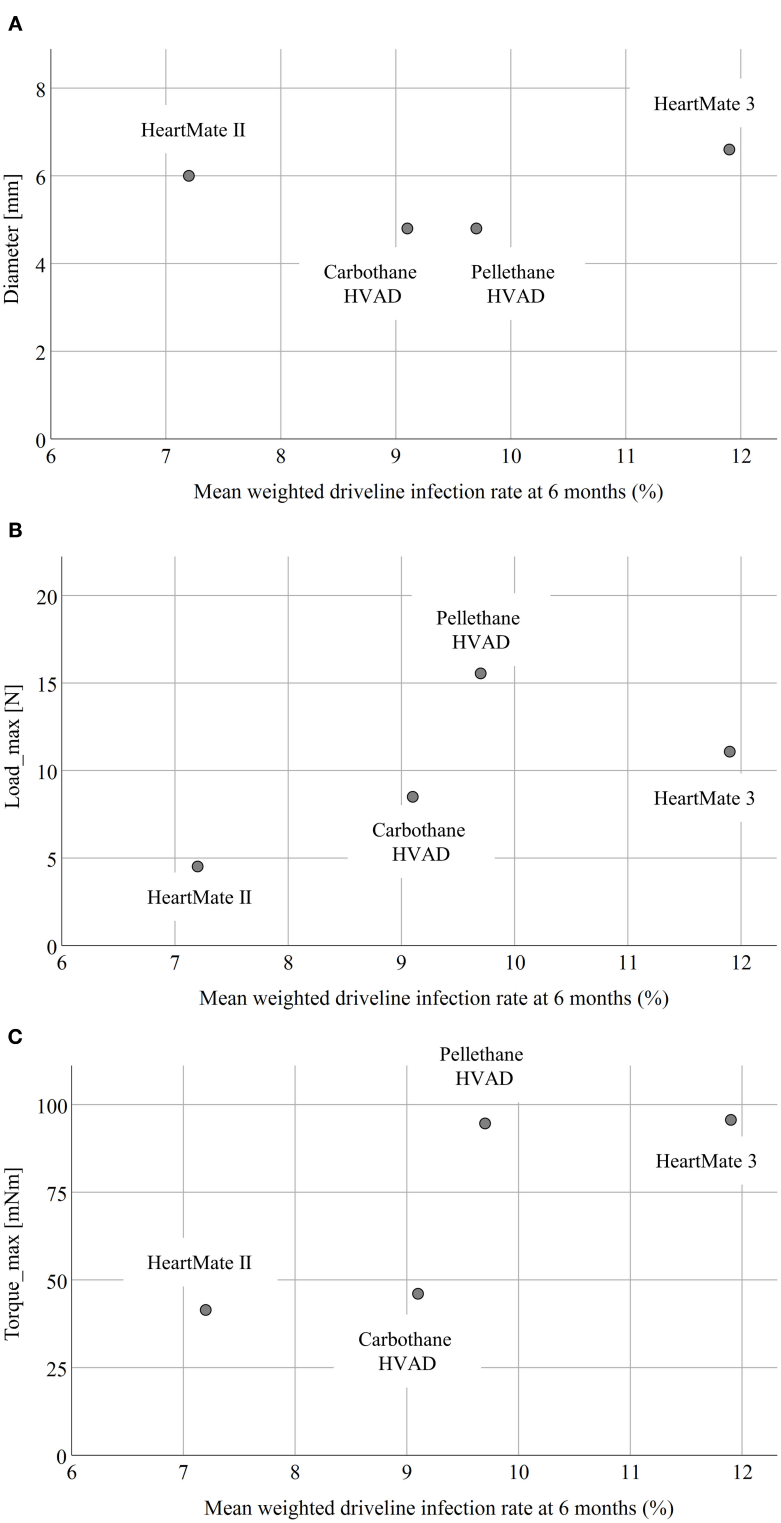
Correlation between the diameter **(A)**, Load_max_ **(B)**, and Torque_max_ **(C)** and the mean weighted driveline infection rate at six months. Load_max_, maximal bending force; Torque_max_, maximal torque.

Figure 5 summarizes the relationships between the mechanical characteristics of the four different drivelines from the *ex-vivo* experimental study and the mean weighted DLI rates at 6 months. No relevant association between the mean weighted DLI rate and the driveline diameter (Figure 5A) or the Load_max_ of the three-point bending test (Figure 5B) was found, respectively. There was an apparent relationship between Torque_max_ of the torsion test and the mean weighted DLI rate (Figure 5C); The HeartMate II driveline had the lowest Torque_max_ and lowest DLI rate, whereas the HeartMate 3 driveline had the highest Torque_max_ and highest DLI rate.

## Discussion

**Figure 6:**
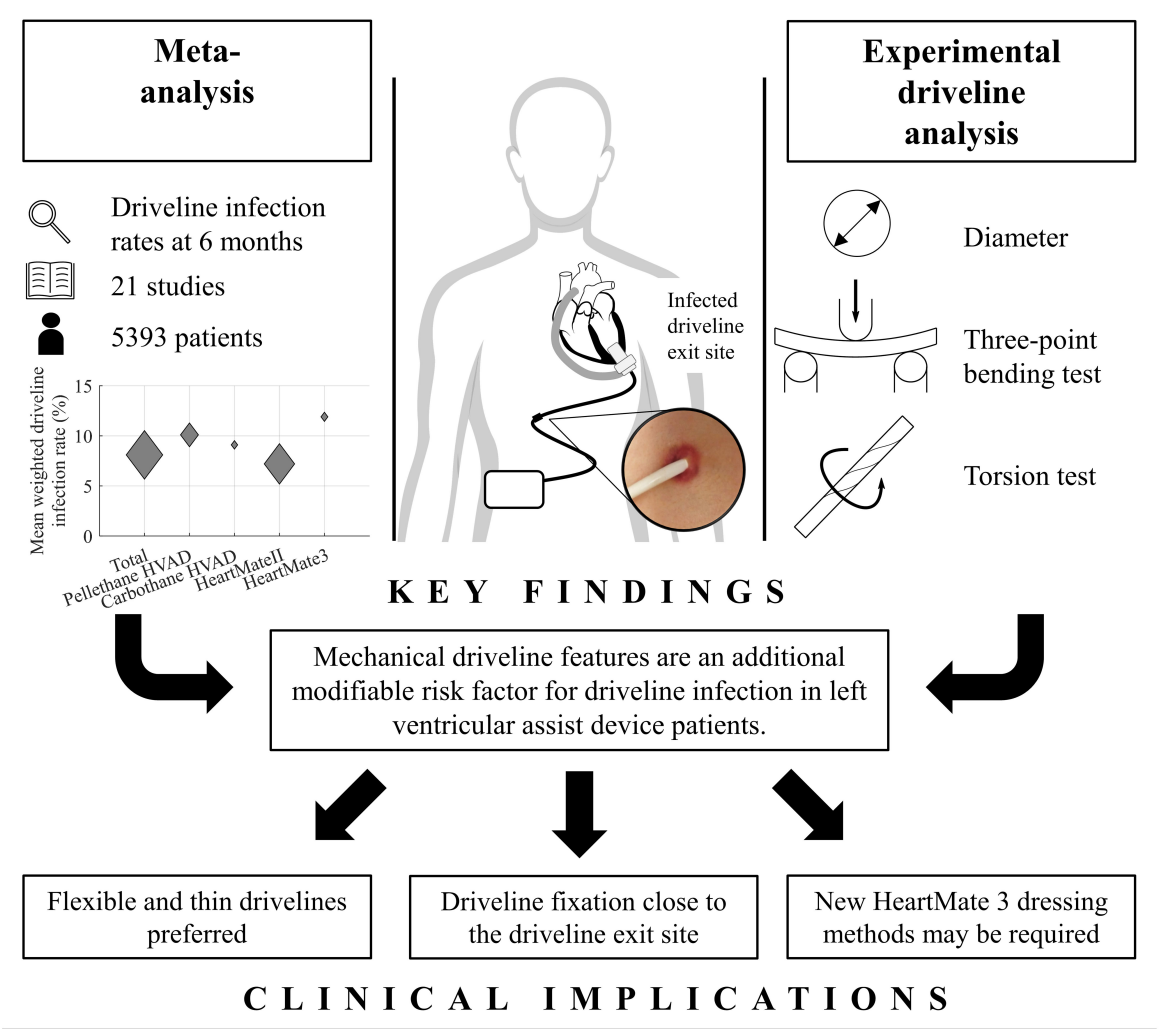
Graphical abstract summarizing the *in-vivo* and *ex-vivo* analysis of mechanical driveline features as a risk factor for driveline infection.

DLI is one of the most common adverse events in the early and late phases after LVAD implantation (1). The development of DLI is multifactorial, with several reported non-modifiable risk factors like DM (10), age (12, 16, 28) or exposed velour (10, 14–16), and, on the other hand, modifiable risk factors (29), such as BMI (10–13), patient lifestyle and activity following hospital, discharge and the associated increase in trauma at the DLES (21). To the best of our knowledge, only one other study has reported the correlation between mechanical driveline features and DLI rates of LVAD-patients (8), but data for contemporary devices are missing. Therefore, the aim of this study was to quantify and compare device-specific driveline characteristics of the HVAD, HeartMate II, and HeartMate 3 as an additional modifiable risk factor associated with DLI, both *in-vivo* and *ex-vivo* (Figure 6).

As previously reported (20), DLI peaks 6 months after LVAD implantation. In this meta-analysis, we found a DLI rate of 8.1% at 6 months, making DLI one of the major adverse events after LVAD implantation. The mean weighted DLI rate was highest with the HeartMate 3 (11.9 %), compared with the Pellethane HVAD (10.1%), the Carbothane HVAD (9.1%), and the HeartMate II (7.2%). Therefore, regardless of patient demographics and center-specific DLES care protocols, the HeartMate II may have positive mechanical driveline features compared to other commercially available LVADs. The approaches for the development of the four contemporary LVAD drivelines investigated in this study are diverse, and different materials are used. Whereas the HeartMate II driveline consists of a soft silicone-based outer layer enveloping an inner jacket made of polyurethane wrapped around a fiber core made of polyethylene (30), the HeartMate 3 driveline has a silicone-based outer layer wrapped around a fiber layer of braided aramid enveloping a polytetrafluoroethylene layer (4). HVAD drivelines consist of an inner silicone lumen enveloped by either Pellethane® or with the new Carbothane® design (23).

Consequently, our *ex-vivo* experimental study showed significant differences in driveline stiffness between all devices (*p* < 0.001) as assessed by the Load_max_ of the three-point bending test (Figure 4A). Even though there was no obvious relationship between Load_max_ and the mean weighted DLI rate, the HeartMate 3 driveline with the largest diameter had the highest DLI rate (Figure 5B). This is in accordance with the findings of Imamura et al., who reported that the HeartMate II driveline had only 20–25% of stiffness and a smaller diameter compared to other devices (EVAHEART, and DuraHeart) and the highest DLI-free rate among those three devices (8). However, the key finding of this study was the hypothesis-generating apparent relationship between higher Torque_max_ of the torsion test and the increased DLI rates (Figure 5C). Therefore, this parameter seems to be a crucial marker for further technical improvements, as driveline torsion is a frequent event in the daily life of LVAD patients, potentially exerting additional force on the DLES and thus leading to trauma-induced DLI as the adherent interface between the velour of the internal part of the driveline and the patient's tissues is critical for the protection against entry of microorganisms and subsequent infection (31). These findings could be relevant to clinical practice, as mechanical features are a modifiable risk factor and exploring more flexible and thinner drivelines would be a simple means to prevent DLI. Based on our *ex-vivo* results and in relation to the clinical DLI rates resulting from the meta-analysis, the most important feature of a LVAD driveline seems to be high flexibility (in terms of low Torque_max_), followed by low stiffness (Load_max_), and minimal thickness (diameter). Although the Medtronic HVAD was recently withdrawn from the market, the development of the new Carbothane driveline appears to be the first step in the multi-faceted strategies to reduce DLI and, by extension, a risk factor for one of the most feared and devastating complications during LVAD support—stroke (7–9). Since the relationship between driveline mechanical properties and DLI rates appears moderate, driveline features are certainly not the “only” risk factor, but are definitely a previously unknown additional factor in DLI development. Therefore, the technical improvement of the mechanical properties of drivelines or even the elimination of them by transcutaneous energy transfer systems (32) should be a high priority in the future development of LVADs. In addition, the development of transcutaneous energy transfer systems will make disappear the need for periodic driveline repairs or for exchanging the HeartMate 3 modular cable—which was necessary in 50% of long-term patients as their active lifestyles caused the cable to deteriorate (33). Thus, the HeartMate 3 modular cable is both a curse and a blessing—the connector enables these necessary driveline exchanges, but this design feature may also be the reason for the higher DLI rates as the rigid modular connector might apply additional traction on the DLES compared to the other devices. Therefore, the results of our study lead to the hypothesis that the overall HeartMate 3 driveline design, including modular connector, is unfavorable, but the findings contrast with the MOMENTUM 3 final report (34), which found no significant but numerically higher 2-year DLI rates with HeartMate 3 (23.3%) vs. HeartMate II (19.4%).

Finally, it should be mentioned that with the HeartMate 3 as the only commercially available LVAD, new DLES dressing methods may be required, including additional binders or anchoring devices (4), e.g., to fix the driveline and the modular connector in a U-shape directly as close as possible at the DLES as well as the rigid connector on the skin to minimize driveline movement and trauma to prevent DLI. The design of next-generation LVAD peripherals should therefore possibly have a combination of external helix pump cable from the controller to the modular connector to absorb additional forces, followed by the most flexible and thin driveline possible to the DLES and implanted pump.

### Limitations

This study has limitations that should be considered when interpreting the results. The meta-analysis was limited to 21 articles (35–47), including only one multicenter study that reported DLI rates of the Carbothane HVAD. Differences in study design, patient characteristics and selection, and center specific DLES care protocols might vary between centers, so we cannot exclude previously reported factors affecting the occurrence of DLI at all. In addition, experimental mechanical testing was limited to a rather modest number of drivelines (*n* = 30), including new and clinically used ones without velour cover or modular driveline connectors (HeartMate 3). The effects of chemical and physical aging of drivelines used *in-vivo* on stiffness were not investigated in this study.

## Conclusion

Device-specific mechanical features of the driveline are an additional modifiable risk factor for the development of DLI and may influence clinical outcomes of LVAD patients.

## Data Availability Statement

The raw data supporting the conclusions of this article will be made available by the authors, without undue reservation.

## Author Contributions

MK, MS, and TS developed the concept and design, performed the statistical analysis, and funding was secured by DZ, GL, HS, and TS. MK and TS drafted the article. MK, A-KS, JR, DW, and CM collected the data. All authors performed critical revision of the article and approved the final version.

## Conflict of Interest

TS has served as a consultant and advisor for Medtronic Inc. and Abbott Inc. and has received research grants from Medtronic Inc. and Abbott Inc. DW has served as a proctor and advisor for Medtronic Inc. and Abbott Inc. HS has served as an advisor for Medtronic Inc. and has received research grants from Medtronic Inc. DZ has served as a proctor, advisor, and speaker for Medtronic Inc., Abbott Inc., Berlin Heart, Edwards, Abiomed, and has received research and travel grants from Medtronic Inc. and Abbott Inc. The remaining authors declare that the research was conducted in the absence of any commercial or financial relationships that could be construed as a potential conflict of interest.

## Publisher's Note

All claims expressed in this article are solely those of the authors and do not necessarily represent those of their affiliated organizations, or those of the publisher, the editors and the reviewers. Any product that may be evaluated in this article, or claim that may be made by its manufacturer, is not guaranteed or endorsed by the publisher.

## Abbreviations

ANOVA: analysis of variance
BMI: body mass index
DLES: driveline exit site
DLI: driveline infection
IQR: interquartile range
LVAD: left ventricular assist device
DM: diabetes mellitus
DT: destination therapy.
