# Supplementary material for: Driveline Features as Risk Factor for Infection in Left Ventricular Assist Devices: Meta-Analysis and Experimental Tests
Source: Front Cardiovasc Med. 2021 Dec 16;8:784208. doi: 10.3389/fcvm.2021.784208 (PMC8716483; doi:10.3389/fcvm.2021.784208)
Supplement: Supplementary file 1 [file Data_Sheet_1.PDF]

Supplementary Material

Supplementary Table S1: Summary of baseline characteristics of the studies included in the meta-analysis. Exit site tunneling: With the conventional technique the driveline penetrates the skin after a short subfascial course directly under the right or left costal arch. The double tunnel technique (8, 41) consists of a long C-shaped subfascial route ending up in a skin incision at the abdominal wall near the midline pointing to the left side of the patient. Triple tunneling (8, 47) including an additional skin incision below the umbilicus with the aim of lengthening the subfascial route of the driveline, and the driveline is turned leftwards into the subfascial layer of the rectus muscle to redirect its exit to the left side via a small incision in the median lower abdomen. Ref., reference number; DLI, driveline infection; INTERMACS, Interagency Registry for Mechanically Assisted Circulatory Support. BMI, body mass index; DT, destination therapy.

**First author, year**	**Ref.**	**Study period**	**Device**	**DLI rate (%)**	**INTERMACS**	**DM**	**Age**	**BMI (kg/m^2)**	**DT**	**Gender (Male)**	**Exit site tunneling**	**Velour**	**Study design**
Bejko et al. 2018	(35)	January 2009 – October 2016	Pellethane HVAD	12.0	1: 41.0%<br>2: 39.0%<br>3: 15.0%<br>4: 5.0%	20%	53 (41-59)	NR	NR	80%	NR	NR	retrospective, single center
Bomholt et al. 2011	(36)	March 2006 – April 2010	HeartMate II	29.0	NR	16%	43 (24-55)	24.2 (21.1-27.3)	19%	74%	conventional tunneling	NR	retrospective, single center
Fudim et al. 2016	(37)	2009 – 2014	HeartMate II	10.4	NR	NR	NR	NR	NR	NR	NR	velour inside	retrospective, single center
			Pellethane HVAD	9.1									
Goldstein et al. 2012	(38)	June 2006 – September 2010	HeartMate II	7.0	NR	NR	NR	NR	NR	NR	NR	NR	retrospective, multi center

Imamura et al. 2017	(8)	2007 – 2015	HeartMate II	4.0	NR	NR	38 ± 13	20.4 ± 3.5	NR	81%	double or triple tunneling	NR	retrospective, single center
John et al. 2016	(13)	June 2005 – June 2014	HeartMate II	7.2	3.8 ± 1.6	37.0%	57.2 ± 14.2	29.2 ± 7.6	21.0%	81.4%	NR	NR	retrospective, single center
Kiss et al. 2020	(39)	December 2011 – November 2018	Pellethane HVAD	2.9	1: 1%<br>2: 29%<br>3: 49%<br>4: 16%<br>5: 4%<br>7: 1%	NR	53 ± 12 (17-69)	25.6 (17.0-37.2)	11%	85%	NR	velour inside	retrospective, single center
Koval et al. 2014	(40)	October 2004 – September 2011	HeartMate II	11.4	1: 30%<br>2: 20%<br>3: 23%<br>4: 25%<br>5: 1.1%<br>6: 0.55%	40%	54 ± 13.8	28 ± 5.9	29%	80%	NR	velour inside	prospective, single center
Lander et al. 2018	(11)	January 2010 – October 2015	HeartMate II<br>- Group 1<br>- Group 2<br><br>Pellethane HVAD<br>- Group 1<br>- Group 2	 15.7<br>14.2<br><br><br>3.3<br>9.0	1: 23.5%<br>2: 36.6%<br>3: 26.1%<br>4: 2.6%	43.8%	58.1 ± 11.9	29.7 ± 6.23	41.2%	NR	NR	velour inside	retrospective, single center

Maltais et al. 2017	(45)	August 2008 – November 2012	Pellethane HVAD	11.0	NR	NR	NR	NR	NR	NR	NR	NR	retrospective multi center
Matsumoto et al. 2019	(41)	August 2013 – May 2017	HeartMate II	4.0	2: 40%<br>3: 60%	NR	45 ± 13	21.2 ± 4.3	NR	73%	52% conventional tunneling, 48% double tunneling	velour inside	retrospective, single center
Netuka et al. 2015	(42)	June 25, 2014 – November 27, 2014	HeartMate 3	10.0	2: 10%<br>3: 42%<br>4: 40%<br>5: 6%<br>6: 2%	24%	58.9 ± 13.5	27 ± 4.3	46%	90%	NR	NR	prospective, multi center
Numan et al. 2021	(43)	December 2010 – December 2019	HeartMate 3	6.8	1: 6.2%<br>2-7: 80.2%	14.8%	56.0 (14.0)	24.7 (6.1)	NR	65.4%	NR	NR	retrospective, single center
			Pellethane HVAD	9.0	1: 5.0%<br>2-7:66.0%	13.0%	58.5 (13.0)	24.1 (5.6)	NR	68.0%			
Ravichandran et al. 2021	(23)	NR	Carbothane HVAD	9.1	1: 8.0%<br>2: 37.0%<br>3: 38.8%<br>4: 16.2%	42.9%	60.5 ± 8.8	27.9 ± 5.3	100%	76.6%	NR	NR	retrospective, multi center
			Pellethane HVAD	14.2									

Schlöglhofer et al. 2021	(4)	2013 - 2017	HeartMate II	9.6	1: 20.9%<br>2: 16.3%<br>3: 20.9%<br>4: 27.9%<br>5: 2.3%	30.2%	57.5 ± 8.7	26.4 ± 4.2	20.9%	93.0%	conventional tunneling	velour inside	retrospective, single center
			HeartMate 3	27.6	1: 10.3<br>2: 6.9%<br>3: 24.1%<br>4: 51.7%	24.1%	62.4 ± 8.3	27.2 ± 5.0	37.9%	89.7%			
			Pellethane HVAD	13.5	1: 27.9 %<br>2: 16.2 %<br>3: 27.0 %<br>4: 21.6%	35.1%	56.1 ± 11.3	25.5 ± 4.3	27.9%	81.8%			
Schmitto et al. 2019	(44)	June 2014 – November 2014	HeartMate 3	10.0	2: 10%<br>3: 42%<br>4: 40%<br>5-6: 8%	NR	59 ± 13	NR	46%	90%	NR	NR	prospective, multi center
Schmitto et al. 2021	(33)	June 2014 – November 2014	HeartMate 3	14.3	3: 75%<br>4: 25%	25%	59 (52–66)	27.61 (20.28-32.44)	NR	75%	NR	NR	prospective, single center
Stahovich et al. 2016	(46)	NR	HeartMate II	7.0	NR	NR	62 ± 11	NR	NR	92%	NR	80% velour inside	prospective, multi center
Stulak et al. 2013	(31)	February 2007 – September 2011	HeartMate II	6..0	NR	21.0%	54	NR	41%	81%	NR	NR	prospective, multi center

Stulak et al. 2016	(19)	May 2004 – September 2014	HeartMate II	4.9	1: 13.0%<br>2: 22.0%<br>3: 33.0%<br>4-7: 32.0%	35%	59.5 (18.0-82.0)	28.5 (2.0-142.2)	47%	83%	NR	NR	retrospective, multi center
			Pellethane	3.6	1: 11%<br>2: 14%<br>3: 45%<br>4: 30%	36%	58.3 (18.3-76.5)	27.6 (16.3-43.7)	19%	72%			
Yoshitake et al. 2018	(47)	April 2011 - July 2015	HeartMate II,<br>Jarvic 2000	8.3	NR	NR	NR	NR	NR	NR	triple tunneling	NR	retrospective, single center

Supplementary Table S2: Quality assessments and risk of bias* of the studies included in the meta-analysis. *Risk of bias was assessed using the tool in the Cochrane Handbook for Systematic Reviews (24). Risk of bias judgment are “Low”, “Moderate”, “Serious”, or “Critical” risk of bias, with an additional option of “Unclear”.

First author, year	Ref.	Risk of Selection Bias	Risk of Performance Bias	Risk of Detection Bias	Risk of Attrition Bias	Risk of Reporting Bias
Bejko et al. 2018	(35)	Low	Low	Low	Low	Moderate
Bomholt et al. 2011	(36)	Low	Low	Low	Unclear	Low
Fudim et al. 2016	(37)	Moderate	Low	Low	Moderate	Low
Goldstein et al. 2012	(38)	Low	Low	Low	Low	Low
Imamura et al. 2017	(8)	Moderate	Low	Low	Low	Low
John et al. 2016	(13)	Low	Low	Low	Low	Low
Kiss et al. 2020	(39)	Low	Low	Low	Moderate	Low
Koval et al. 2014	(40)	Low	Low	Low	Low	Low
Lander et al. 2018	(11)	Moderate	Low	Low	Low	Low
Maltais et al. 2017	(45)	Moderate	Low	Low	Low	Low
Matsumoto et al. 2019	(41)	Low	Low	Low	Unclear	Low
Netuka et al. 2015	(42)	Low	Moderate	Low	Low	Low
Numan et al. 2021	(43)	Low	Low	Low	Low	Low
Ravichandran et al. 2021	(23)	Low	Low	Low	Unclear	Low
Schlöglhofer et al. 2021	(4)	Low	Low	Low	Low	Low
Schmitto et al. 2019	(44)	Low	Moderate	Low	Low	Low
Schmitto et al. 2021	(33)	Low	Moderate	Low	Low	Low
Stahovich et al. 2016	(46)	Moderate	Low	Low	Low	Low

Stulak et al. 2013	(31)	Low	Low	Low	Unclear	Low
Stulak et al. 2016	(19)	Low	Low	Low	Moderate	Low
Yoshitake et al. 2018	(47)	Low	Low	Low	Unclear	Moderate
